# Can an alert in primary care electronic medical records increase participation in a population-based screening programme for colorectal cancer? COLO-ALERT, a randomised clinical trial

**DOI:** 10.1186/1471-2407-14-232

**Published:** 2014-03-31

**Authors:** Carolina Guiriguet-Capdevila, Laura Muñoz-Ortiz, Irene Rivero-Franco, Carme Vela-Vallespín, Mercedes Vilarrubí-Estrella, Miquel Torres-Salinas, Jaume Grau-Cano, Andrea Burón-Pust, Cristina Hernández-Rodríguez, Antonio Fuentes-Peláez, Dolores Reina-Rodríguez, Rosa De León-Gallo, Leonardo Mendez-Boo, Pere Torán-Monserrat

**Affiliations:** 1 Primary Healthcare Centre Santa Rosa, Catalan Health Institute, Carrer El Cano s/n, 08921 Santa Coloma de Gramenet, Spain; 2 Primary Healthcare Research Support Unit Metropolitana Nord, Institut Universitari d’Investigació en Atenció Primària (IDIAP) Jordi Gol, Carrer Major 49-53, 08921 Santa Coloma de Gramenet, Spain; 3 Primary Healthcare Centre Sanllehy, Catalan Health Institute, Av Mare de Deu de Montserrat 16-18, 08024 Barcelona, Spain; 4 Primary Healthcare Centre Riu Nord-Riu Sud, Catalan Health Institute, Carrer Major 49-53, 08921 Santa Coloma de Gramenet, Spain; 5 Department of Internal Medicine, Fundació Hospital de l’Esperit Sant, Avinguda Mossèn Pons i Rabadà s/n, 08923 Sta Coloma de Gramenet, Barcelona, Spain; 6 Department of Preventive Medicine and Epidemiology, Hospital Clínic, Carrer del Rosselló 138, 08036 Barcelona, Spain; 7 Department of Epidemiology and Evaluation, Hospital del Mar, Passeig Marítim, 25-29, 08003 Barcelona, Spain; 8 Direcció d’Organització i Sistemes, Gerencia Territorial Metropolitana Nord, Catalan Health Institute, Ctra.de Canyet s/n, 08916 Badalona, Spain; 9 Metodology, Quality and Care Evaluation, Metropolitana Nord Primary Care Service, Catalan Health Institute, Badalona, Spain; 10 Primary Care Services Information System, Catalan Health Institute, Avinguda Gran Vía de les Corts Catalanes 587, 08007 Barcelona, Spain; 11 Colorectal Screening Programme Research Group (PROCOLON), Barcelona, Spain; 12 Health Services and Chronic Diseases Research Network (REDISSEC), Barcelona, Spain; 13 Hospital del Mar Medical Research Institute (IMIM), Barcelona, Spain; 14 Grupo emergente de investigación en cáncer (CANCER-AP), IDIAP JordiGol, Catalan Health Institute, Barcelona, Spain

## Abstract

**Background:**

Colorectal cancer is an important public health problem in Spain. Over the last decade, several regions have carried out screening programmes, but population participation rates remain below recommended European goals. Reminders on electronic medical records have been identified as a low-cost and high-reach strategy to increase participation. Further knowledge is needed about their effect in a population-based screening programme. The main aim of this study is to evaluate the effectiveness of an electronic reminder to promote the participation in a population-based colorectal cancer screening programme. Secondary aims are to learn population’s reasons for refusing to take part in the screening programme and to find out the health professionals’ opinion about the official programme implementation and on the new computerised tool.

**Methods/Design:**

This is a parallel randomised trial with a cross-sectional second stage. Participants: all the invited subjects to participate in the public colorectal cancer screening programme that includes men and women aged between 50–69, allocated to the eleven primary care centres of the study and all their health professionals. The randomisation unit will be the primary care physician. The intervention will consist of activating an electronic reminder, in the patient’s electronic medical record, in order to promote colorectal cancer screening, during a synchronous medical appointment, throughout the year that the intervention takes place. A comparison of the screening rates will then take place, using the faecal occult blood test of the patients from the control and the intervention groups. We will also take a questionnaire to know the opinions of the health professionals. The main outcome is the screening status at the end of the study. Data will be analysed with an intention-to-treat approach.

**Discussion:**

We expect that the introduction of specific reminders in electronic medical records, as a tool to facilitate and encourage direct referral by physicians and nurse practitioners to perform colorectal cancer screening will mean an increase in participation of the target population. The introduction of this new software tool will have good acceptance and increase compliance with recommendations from health professionals.

**Trial registration:**

Clinical Trials.gov identifier NCT01877018

## Background

### Epidemiology

In Spain, colorectal cancer (CRC) has the highest incident-rate in both sexes [1], with more than 25,000 new cases diagnosed annually. Approximately 90% of CRC diagnoses occur after the age of 60 and the majority (70%) are sporadic cases. The incidence of CRC in Spain has increased from 6 cases/100,000 inhabitants per year in 1973 to 30.4 cases/100,000 inhabitants per year in 2008, with epidemiological estimates of up to 33,000 cases in 2012 [1]. It is the second leading cause of cancer death in both sexes, after lung cancer in men and breast cancer in women. Mortality rates appear to be levelling off in recent years, most likely due to the improved diagnosis and treatment of this disease [2]. Spain has an average ranking in terms of incidence and mortality compared with other European countries with 5-year-survival rates standing at 54.7% for colon cancer, and 50.2% for rectal cancer [3] and it is estimated that in the next few years, one in 20 men and one in 30 women will develop a CRC before the age of 75 [4].

### Current status of CRC screening in Spain

CRC meets the requirements for the implementation of a screening programme [5]: it has a known natural history based on precursor lesions (adenomatous polyps), represents a public health problem owing to its high incidence and mortality rate, there are effective tests available for the early detection of the illness and its treatment in early stages improves its prognosis with tests widely accepted by the public. The purpose of screening is to reduce disease-specific mortality, with minimal risks of over-diagnosis and over-treatment. The cost-effectiveness of CRC screening programmes has been amply demonstrated, where it is eight times more cost-effective than screening for breast cancer in Spain. There is international consensus on the interest in screening the average-risk population, namely men and women aged 50 and upwards [6-9]. The effectiveness of screening using the faecal occult blood test (FOBT) has been widely demonstrated in randomised clinical trials with a drop in both mortality (15% to 33%), and incidence rates (20%) [10,11]. The current immunological faecal occult blood test (iFOBT), based on the detection of human haemoglobin through specific antibodies, have been established as the technique of choice in different screening programmes implemented in Europe and have replaced conventional methods such as the guaiac method, based on pseudoperoxidase activity of haemoglobin [12-14]. The Council of the European Union recommends the FOBT in men and women aged 50 to 74, every two years [15]. The Spanish Ministry of Health’s National Health System Cancer Strategy promoted the implementation of screening programmes for men and women between 50 and 69 currently covering 14% of the target population, with the aim of reaching 50% by 2015 [16]. These screening programmes implemented in Spain follow the criteria of the European Guidelines for quality assurance in colorectal cancer screening [17] and are coordinated through the network of cancer screening, allowing common methodological approaches to be followed and the availability of compatible information systems to facilitate evaluation and comparison of both the process and the results [18]. The participation of the population in these programmes represents a quality indicator referred to in the European guidelines and is an important pillar for ensuring its effectiveness. Experts consider a minimum uptake of at least 45% is acceptable, but it is recommended to aim for a rate of at least 65%. However, similar to other countries, results from existing programmes in Spain show that the participation of the population is not reaching the recommended objectives, with varying levels of uptake rates (17%–42%), with the exception of the Basque Country (64.3%) [19-23]. It can be generally said that participation has been highest where there is increased primary health care involvement.

### Strategies to increase participation: electronic reminders

Certain randomised clinical trials support the effectiveness of different interventions promoted by primary care to increase participation in CRC screening, although demonstrating differences in their impact and depending on the economic coverage of the tests, screening basal rates, target level and number of interventions [24]. Some of these initiatives eat into resources both in terms of personnel and time, making their reproducibility impractical in certain primary health offices which are becoming increasingly overburdened. Several authors point to new technological strategies to improve the coverage and evaluation of CRC screening [25]. The introduction of specific electronic reminders or alerts in electronic medical records (EMR) has proven effective to increase the practice of different preventive activities, including cancer prevention, with increases of up to 12–14% [26,27]. This increase is most noticeable in centres with greater levels of cohesion, where they already have a computerised medical record system of the population in place. Despite its broad scope and low cost, electronic reminders still remain an under-utilised tool in the healthcare sector [28]. The introduction of reminders aimed at physicians for the promotion of CRC screening with the FOBT remains a controversial matter in terms of its effectiveness in revised literature [29,30]. However, these studies have been carried out in countries that offer a context that is different to ours with regards to the type of programme (population-based/opportunistic), test economic coverage (public/insured), test type (FOBT/colonoscopy) and degree of implementation of computerised medical records in health centres, to name a few. A study has been carried out on how professionals have adhered to executing the electronic reminders, identifying ways of making this easier, such as limiting the number of reminders, integrating them into the medical visit and facilitating follow-up technical support. Strategies to address barriers identified such as the allocation of responsibility among medical and nursing staff, visibility of alerts or the existence of a feedback mechanism on its use have also been proposed [31,32].

### Involvement of primary health care in the CRC screening programme in Catalonia

A population-based programme for the early detection of colorectal cancer in Barcelona started in 2009 [33]. Patients receive a nominal mail issued centrally from the programme’s offices, inviting them to participate. The quantitative iFOBT screening test is used, which is performed at home and is distributed at the community pharmacy offices attached to the colorectal cancer screening programme (CRCSP). Participants registering a negative result are invited to participate again in two years. Positive cases are referred by telephone to a specific consultation for evaluation with a colonoscopy. If the colonoscopy comes back normal, the patient is invited to repeat the iFOBT in ten years time. In the event of endoscopic findings, the patients are referred for follow-up in primary care in the case of adenoma, or specific consultations, the CRC High Risk Clinic or the CRC unit, depending on the pathology found. The primary care health professionals are informed of the implementation of the circuit in the population they are treating and the importance of its promotion, in a specific session provided at each primary care centre (PCC). Certain sections of the different Primary Care medical scientific societies have expressed their disagreement with the current approach to CRC screening programmes that do not directly involve the primary care provider.

The magnitude of CRC as a public health problem, the less than ideal participation reflected in the different programmes that have recently been implemented in Spain, international evidence of the benefits that the involvement of primary care professionals has for the participation in screening programmes and the low levels of literature available nationwide, has prompted us to perform this study.

### Objectives

The main objective is to evaluate the effectiveness of an electronic alert in patients’ EMR to increase their participation in a population-based CRC screening programme in Barcelona.

The secondary objectives are:

1. To discover the reasons for non-participation in a population-based CRC screening programme

2. To find out the opinion of health professionals about the electronic alert (Colo-alert)

3. To find out the opinion of health professionals about the population-based CRC screening programme.

## Methods/Design

### Methodology

The COLO-ALERT study is a randomised clinical trial comparing standard clinical practice (control group) in relation to the activation of an electronic alert in EMR (intervention group) of patients in primary care to promote and increase participation in a population-based CRCSP. It also comprises a second cross-sectional and observational stage.

#### Stage 1: COLO-ALERT a randomised clinical trial

*Design*: A parallel randomised clinical trial.

*Setting*: Eleven PCC, representing nine care teams, in urban areas, from the Primary Care Services of Barcelona, of the Catalan Health Institute.

### Participants

People involved to participate in the CRCSP and their respective primary care health professionals from the centres to which they are assigned. Table 1 shows the criteria for CRCSP inclusion and exclusion.

**Table 1:** Inclusion and exclusion criteria for the programme for early detection of colorectal cancer in Barcelona

**Programme for early detection of colorectal cancer in Barcelona**	
**Target population**	Men and women aged 50–69 included in Catalonia’s Registro Central de Asegurados
**Exclusion criteria**	Personal history of colorectal cancer
	Suspicion symptoms of colorectal cancer: blood in stools, change in bowel habits for more than 6 weeks, unexplained weight loss or fatigue or persistent abdominal discomfort
	Family history of colorectal cancer: 2 first-degree relatives (parents, siblings or children) diagnosed with colorectal cancer or one first -degree relative diagnosed with colorectal cancer before the age of 60
	Family history of familial adenomatous polyposis and other polyposis syndromes, or Lynch syndrome
	History of colorectal disease susceptible to specific monitoring (ulcerative colitis, Crohn’s disease, colorectal adenomas)
	Terminal illness or serious illness or disability that would contraindicate further study of colon
	History of total colectomy
	Death
	Colorectal examinations performed in the last 5 years
	Address error

Inclusion criteria:

– *For patients*:

a) men and women aged between 50 and 69 invited to participate in the CRCSP

b) to be assigned with a primary care physician (PCP) at one of the study centres.

– *For health care professionals*: physicians or nurses working at the study centres.

**Figure 1:**
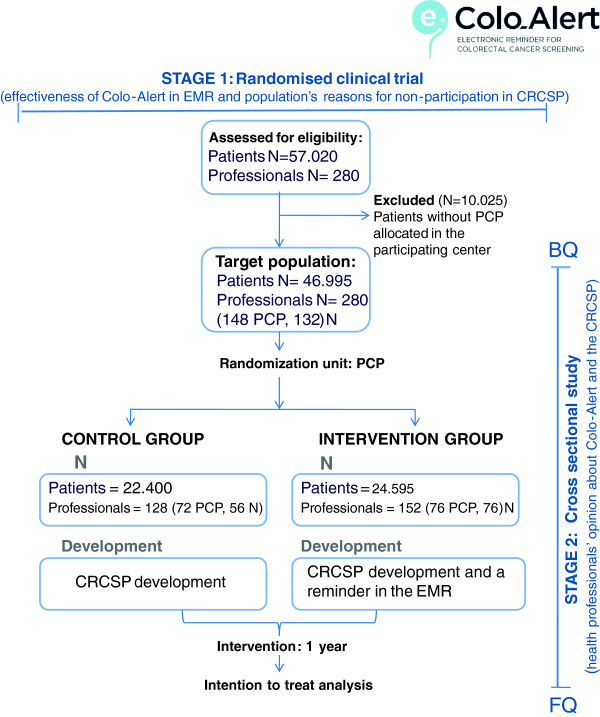
**Flow chart of the COLO-ALERT Study.** PCP: Primary care physician, N: Nurse, CRCSP: Colorectal cancer screening programme, EMR: electronic medical record, BQ: baseline questionnaire, FQ: final questionnaire.

Figure 1 shows the flow chart for the study.

### Recruitment of participants

*Recruitment*: Whenever the population centre, is invited to participate at first round of CRCSP, from July 2011 to May 2012, the heads of coordination and management of the programme conduct a training session addressed at all staff in the centre about CRC screening and the established circuit. The previous minutes of this meeting are reserved for the research team for the presentation of the study, inviting centres to participate. If a centre agrees to participate, all its health professionals and their respective patients that may participate in the CRC screening programme are included in the study. The follow-up period will be one year.

*Collection of data and information sources*: The heads of CRCSP are asked to provide a list of the personal identification code of patients invited to participate in the study setting at the beginning of the study. They are then encrypted from the Catalan Health Institute’s Primary Care Services Information System and the physicians and nurses assigned in the participating centres are identified. Randomisation and allocation of the study groups is then performed. At the end of the study, CRCSP will provide a list with the result of the participation of patients. The patient information is obtained based on the personal identification code, from the EMR, the data provided by the Primary Care Services Information System, and the data provided by the heads of CRCSP, creating a unified database for the purpose of linking the information.

*Randomisation*: The PCP is the unit of randomisation. The allocation of the participating physicians to the control or intervention group is carried out, by the statistician of the study, through a stratified random sampling by centre, allocating 50% of the physicians to the control or intervention group, respectively. The nurses are allocated to the control or intervention group according to the study group of the PCP that they share patients care with. Patients are allocated to the control or intervention group according of the study group of their PCP (Figure 1).

*Blinding*: Given the nature of the intervention, it is not possible to carry out blinding of the health professionals randomised to the intervention group, neihter of the statistic responsible for the data analysis. However, given the objectivity of the primary outcome, we do not believe the result could be influenced by this fact. Patients are unaware of study group that they have been assigned to and they have no access to the EMR. In addition, the CRCSP representative responsible for obtaining the data on the primary outcome does not know what study group the invited population was allocated to.

### Interventions and procedures

*Control group*: Includes all health professionals randomised as a control group and their assigned patients that have been invited by the CRCSP They are following the procedures of the Barcelona CRCSP functional plan [33].

**Figure 2:**
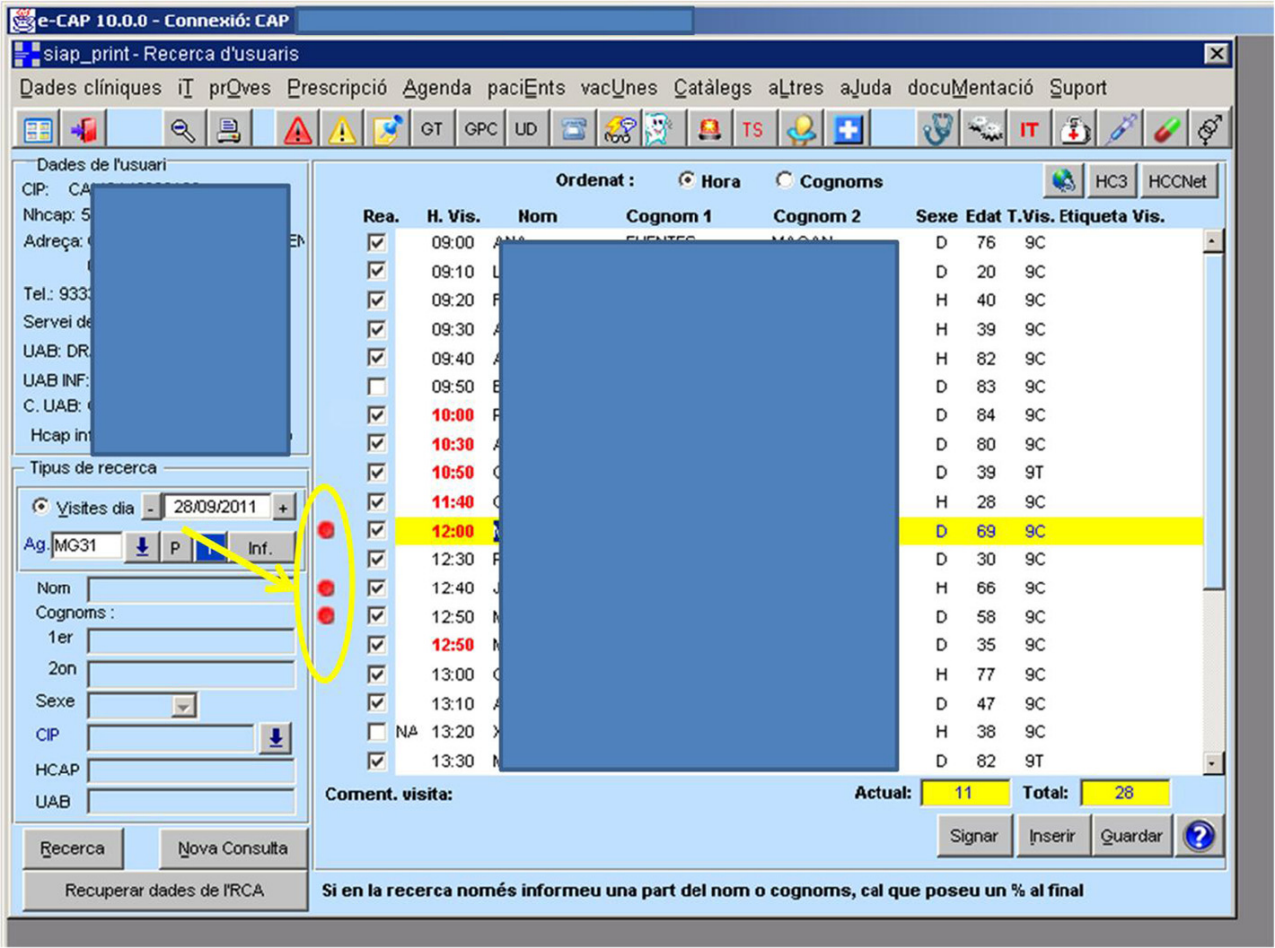
**Study electronic alert.** Specific icon (red dot) in front of the patient’s name on the list of appointments scheduled for the day.

*Intervention group*: Includes all health professionals randomised as the intervention group and their assigned patients that have been invited by the CRCSP. They are following the CRCSP functional plan described above along with the activation of an electronic alert linked to the subjects’ personal identification code. Intervention consists of the introduction of an alert in the patients’ EMR, appearing as a specific icon, in the agenda of patients with appointments for that day, identifying those subjects who have been invited to participate in the CRCSP (Figure 2). It is intended for physicians and nurses, to promote CRC screening actively during a synchronous medical visit with the patient, by means of a structured brief recommendation to this effect. They are also invited to complete the data collection sheet designed for the study and also entered into the EMR. Health professionals from the intervention group receive a specific training session during which they are explained the features of the electronic alert and how it works.

The alert is activated once the population belonging to the centre joins the screening program. Once completed, it is then deactivated, with a maximum period of one month to refine it. In any other case, it remains visible at each patient’s visit, until the end of the study (1 year).

### Outcomes

#### Primary outcome

● CRC screening status (provived by CRCSP)

– Participation: performance of the iFOBT, by invitation from CRCSP during the study period (1 year).

– Non-participation: where the iFOBT is not performed, by invitation from CRCSP during the study period.

– Exclusion: detection of any exclusion criteria through the CRCSP screening process.

#### Secondary outcomes

*Patient profiles*: Data obtained from the EMR at the beginning of the study.

Personal identification code, age, sex, socio-economic deprivation index [34], body mass index, smoking (non-smoker, smoker, ex-smoker), alcohol consumption (non-drinker, low risk, high risk), clinical risk group (1: healthy, 2: acute illness, 3: minor chronic illness, 4: multiple minor chronic illness, 5: Dominant chronic illness; 6: two dominant chronic illnesses 7: three dominant chronic illnesses; 8: neoplasms; 9: catastrophic illness), frequency (number of visits to the PCC during the study period).

*Related to the execution of the electronic alert* (specific data collection sheet in the EMR):

Revised alert (completion of the study data collection sheet included in the patients EMR by the health professionals) and date. Includes variables: oral informed consent, participation in the screening programme using the iFOBT undertaken prior to the physician’s medical appointment at the PCC, reasons for non-participation, presence of exclusion criteria specific to the screening program, intention to participate in the program.

*Related to the screening result*: Date and result of performance of iFOBT provided by CRCSP at the end of the study.

*Sample size*: The assigned population at the study centres aged between 50 and 69 is comprised of about 60,000 inhabitants. An estimated 90% of subjects will be invited to participate in the CRCSP. In the event of a low screening uptake rate (30%), accepting an alpha risk of 0.05 and a beta risk less than 0.05 in a bilateral contrast, 19,181 patients are needed in each group to detect a difference of 1.7 percentage points between the control group and the intervention group.

#### Stage 2: cross-sectional COLO-ALERT

*Design*: Descriptive cross-sectional study.

*Setting*: The PCC included in stage 1.

*Participants*: All health professionals (physicians and nurses) from a PCC who have given their consent to participate.

*Data collection*: For the second cross-sectional stage of the project, information on health professionals will be collected based on two self-administered on-line questionnaires. An e-mail will be sent to the professional’s work e-mail address, facilitated by the administration of each centre, with a link to complete the survey and personal access codes. A reminder will be sent to the professionals who have not completed the form one week later. The baseline questionnaire will be sent at the beginning of the study, collecting data on the profile of the professionals. The final questionnaire will be sent at the end, in order for the professionals to evaluate the CRCSP and the newly-introduced software tool.

### Variables

#### Baseline questionnaire

Age, sex, profession and specialty, year of graduation, average work load, PCC, knowledge about colorectal cancer screening (questions based on the general recommendations of the National Clinical Practice Guideline will be included on: epidemiology, risk stratification, effectiveness, testing, target population, endoscopic surveillance intervals) [8].

#### Final questionnaire

The information will be categorised in nominal or numeric variables, using a Likert scale, which includes the following areas:

– Official program: Information received, process operation, involvement of primary care professional, recommendations for improvement (open).

– Electronic alert: utility, operation, use, recommendations for improvement (open).

### Statistical analysis

Once the data has been filtered, a standard deviation, median and univariate descriptive analysis will be carried out for the quantitative variables with normal and median distribution and inter-quartile range for quantitative variables with non-normal distribution and frequency and percentage for qualitative variables. Analysis will be carried out by intention-to-treat (screening), where any patients who are lost at the end of the year that the intervention takes place due to changes of address, institutionalisation, or death will be considered as absent from the screening. The same analysis will be later carried out exclusively on patients who have completed the intervention, and the results of these two methods will be compared at the end. The bivariate relationship between final participation in the CRCSP and each of the variables that define the profile of the patients will be evaluated using the *t*-test to obtain the mean difference in the case of a quantitative variable and categorical and with the Chi-square test to compare proportions in the case of two qualitative variables. The frequency and percentage of patients screened per group will be calculated and the two proportions compared using the Chi-square test (main objective). A multiple logistic regression model will be set, where the screening will have been completed by the dependent variable and the group and other variables that define the profile of patients as independent variables. This will allow us to discover which patient characteristics are associated with participation in the screening program. Finally, the evaluation survey variables will be described for the professionals using a univariate descriptive analysis, as well as a bivariate analysis where associations will be evaluated two by two between survey variables and the variables that define the profile of the professionals (secondary objective). All statistical tests will be performed with a bilateral confidence level of 95%. The collected data will be analysed with the Stata statistical programme version 12.1.

### Ethics and confidentiality

The researchers undertake to respect the rules of Good Clinical Practice and the Guidelines of Good Practice in Research of the Primary Care research institute (IDIAP) Jordi Gol, the requirements of the Declaration of Helsinki and the general ethical clauses, particularly those regarding the right to privacy, anonymity and confidentiality. This project has the approval of the Jordi Gol Primary Care Research Institute’s Ethics and Clinical Research committee (P10/31). As our objective is to evaluate the real impact of intervention in standard clinical practice, the health care professional participating in the study will be informed personally of their participation in a research project that involves the activation of reminder systems to promote population screening for colorectal cancer. They will then receive detailed information about the study in a specific session at their health centre. The acceptance of a primary care centre’s participation in the study is decided on jointly by the team of family medicine and nursing professionals and all of them are included for randomisation. We do not request written informed consent from the healthcare professional participating in the study nor a minimum number of revised alerts, to avoid bias of highly motivated professionals and in order to simulate the actual conditions of standard clinical practice as much as possible. Similar experiences are cited in the reviewed literature on the subject [35]. The participating patients will be informed verbally about the study and their oral consent to participate will be recorded in an electronic data collection sheet, which will be entered into their medical record.

## Discussion

Numerous organisational and cognitive factors influencing inadequate coverage in the actual practice of mass screening for CRC. These factors are derived from both patients and healthcare and administration professionals. The low participation is partly due to a lack of awareness of both the illness itself and the early detection programmes, but also to the existence of barriers for the conduct and results of the tests. It is essential to inform the population in question about the magnitude of CRC, the importance of early detection, the benefits and risks of participating in this type of programme and the need to coordinate and involve the different health professionals and institutions that participate directly or indirectly in a screening program.

Direct recommendation by the family physician has been described as one of the strongest predictors for the performance of CRC screening, while the non-involvement of this level of care in the recommendations is one of the main reasons for it not being carried out [36,37]. Data published in our sector show that 89% of subjects would accept CRC screening if their primary care physician or nurse suggested it; a percentage that is very different from the data presented in the current population programmes [38]. On the other hand, most of the eligible population in countries with a long history of CRC screening have shown that they have never received such a recommendation [39]. The reality of the primary care professional’s offices with an overload of care, preventive and bureaucratic tasks influence the poor level of recommendation for CRC screening in the target population attending the clinic for other reasons.

There are clinical studies in place that support the effectiveness of electronic reminders in clinical practice. Nease et al. found a significant increase of 9% in terms of the performance of FOBT, despite a low rate of revision for electronic alerts (30%) [29]. Sequist et al. found an increase in screening rates in those patients who attended the surgery on more than two occasions during the study period, although the difference was not significant, in part due to very high baseline screening rates already in existence and also owing to the fact that the colonoscopy was the test of choice of physicians when recommending screening, with an uptake rate of only 50% of patients [40]. Nease and Sequist evaluate the acceptance and integration of reminders into medical practice with a good general level of acceptance. However, there are certain limitations, such as the moderate suitability of alerts activated in patients considered candidates for screening, possibly generating a tendency to wilfully overlook the reminders, or see them as an interference in the course of medical visits owing to care overload.

The following are worth mentioning as possible limitations of this study:

The selection of the CRCSP target population is based on data from patients included in Catalonia’s Registro Central de Asegurados (Registry of Users of the Catalan Health-care System). The percentage of patients on this register that are assigned to a PCC, and would therefore be invited to participate in the programme when it starts screening, but in actual practice reside at another address or attend another centre, accounts for 19% of the study population, much higher than the average of Catalonia, which was 8.1% according to 2012 figures. Alerts cannot be activated in the medical records of these subjects, as they do not have a physician assigned to the centre that will be participating in the screening program. This may involve a certain level of selection bias, but there is no reason to believe that this population attending a different centre to the one they are assigned may have some distinguishing feature in relation to the study groups and in any case, the control and intervention groups are distributed on a random basis.

While the intervention is directed at the population receiving care, this represents the majority of the assigned population as the duration of the intervention is one year. In 2011, 69% of patients aged between 50 and 69 made at least one visit to their centre involved in the study, where the overall average in Catalonia stands at 71%.

Losses during follow-up: changes of address, institutionalisation or death may occur during the course of the study. Any of these scenarios will be considered as the screening having not taken place.

External validity: This involves a study of urban population, but since the use of EMR is used across the board in primary care in Catalonia, no differences in the effectiveness of electronic reminders are forecast according to the scope of work.

Contamination between professionals: Since the unit of randomisation is the physician, certain contamination could occur between centre professionals. In order to minimise this, a training session on the computerised tool exclusively for professionals in the intervention group is provided. The decision to randomise by medical professional was made by significant socio-demographic differences existing in the reference population of the study centres and by the differences in basal participation found in other centres already screened in the same field, exceeding 10% on occasion.

The CRC screening programmes in Spain are population-based, providing access to the target population, and biennial iFOBT is the test that has been selected, which has shown better levels of acceptance and participation among the population. On the other hand, health professionals from the PCC have a long history in the use of EMR, with universal coverage of the population. In light of this, we are considering the introduction of a specific reminder in the primary care EMR of the target population for an early detection programme for CRC. The healthcare professional will provide the identification and recommendation directly to the patient when he/she attends his/her health professional for any other reason, resulting in increased participation, and thus improving its cost-effectiveness and quality indicators.

## Abbreviations

CRC: Colorectal cancer; FOBT: Fecal occult blood test; iFOBT: Immunochemical fecal occult blood test; EMR: Electronic medical records; CRCSP: Colorectal cancer screening programme; PCC: Primary care centre; PCP: Primary care physician; IDIAP: Primary care research institute.

## Competing interests

The authors of this manuscript have no competing interests.

## Authors’ contributions

CGC, PTM contributed to formulating the research question. CGC, PTM, IRF, CVV, MVE, MTS, LMO, JGC, ABP contributed to the study design. CGC is the coordinator of the investigation. CGC, IRF, CVV, MVE are responsible for conducting de trial. MVE, CVV, CGC, MTS, IRF, JGC, ABP elaborated the patient data collection sheet and DRR, AFP, RLG managed its introduction and surveillance in the EMR. MVE, CVV, CGC, MTS, IRF, JGC, ABP also elaborated the two questionnaires addressed to health care professionals and AFP performed the on-line version of both. CGC, IRF conducted the training session on the intervention’s development. LMB contributed to the design, activation and refinement of the alert introduced in the EMR. CHR is responsible for the data collection and management of the screening programme databases. LMO supervised the methodology of the protocol of investigation and will be responsible for the treatment of the data and statistical analysis. All authors contributed to and approved the final manuscript.

## Authors’ information

CGC, IRF, MVE, CVV are family physicians from the Catalan Health Institute and belong to the emerging group of clinical research in Cancer from the Jordi Gol Primary Care Research Institute (IDIAP). PTM is the coordinator and LMO is the statistician from the research support unit of the primary care research institute for the northern metropolitan area of the Catalan Health Institute. MTS specialises in digestion and is director of internal medicine department at the Espíritu Santo Hospital. ABP is a specialist physician in preventive medicine and public health, with a doctorate in public health, and belongs to the research network of health services in chronic diseases (REDISSEC) and to the Hospital del Mar research institute (IMIM). CHR is the colorectal cancer screening programme data manager. ABP and CHR work in the epidemiology and evaluation service of the Hospital del Mar. JGC is a specialist physician in preventive medicine and public health, working in the department of preventive medicine and epidemiology at the Hospital Clinic of Barcelona. ABP, CHR and JGC belong to the colorectal screening programme research group (PROCOLON). RLG is a family physician at the Catalan Health Institute and leader in his field from the EMR software program. DRR works in the department of methodology, quality and evaluation for the northern metropolitan area of the Catalan Health Institute. AFP is a computer programmer for the northern metropolitan area of the Catalan Health Institute. LMB is a specialist in public health and is part of the Catalan Health Institute’s Primary Care Services Information System.

## Pre-publication history

The pre-publication history for this paper can be accessed here:

http://www.biomedcentral.com/1471-2407/14/232/prepub
